# The Abilities of Salidroside on Ameliorating Inflammation, Skewing the Imbalanced Nucleotide Oligomerization Domain-Like Receptor Family Pyrin Domain Containing 3/Autophagy, and Maintaining Intestinal Barrier Are Profitable in Colitis

**DOI:** 10.3389/fphar.2019.01385

**Published:** 2019-12-02

**Authors:** Jiuxi Liu, Jiapei Cai, Peng Fan, Naisheng Zhang, Yongguo Cao

**Affiliations:** Department of Clinical Veterinary Medicine, College of Veterinary Medicine, Jilin University, Changchun, China

**Keywords:** salidroside, colitis, NLRP3 inflammasome, autophagy, intestinal barrier, inflammation

## Abstract

Salidroside (Sal), as a major glycoside extracted from *Rhodiola rosea* L., has exhibited its mighty anti-aging, anti-oxidant, anti-cancer, anti-inflammation, and neuroprotective effects in many diseases. Recently, it has showed its protective effect in colitis mice by activating the SIRT1/FoxOs pathway. Whereas, it is not known whether Sal has other protective mechanisms on dextran sulfate sodium (DSS)-induced colitis in mice. In this study, we investigated the protective effects and mechanisms of Sal on DSS-induced colitis in mice. The results demonstrated Sal was a competent candidate in the treatment of ulcerative colitis (UC). Sal remitted DSS-induced disease activity index (DAI), colon length shortening, and colonic pathological damage. Simultaneously, Sal alleviated excessive inflammation by reversing the IL-1β, TNF-α, and IL-10 protein levels in DSS-treated mice. Western blot analysis revealed that Sal inhibited p65 and p38 activation together with peroxisome proliferator-activated receptor (PPARγ) up-regulation. In addition, Sal skewed the imbalanced activation of nucleotide oligomerization domain-like receptor family pyrin domain containing 3 inflammasome and autophagy contributing to colitis recovery. The damaged intestinal barrier induced by DSS was also alleviated along with plasma lipopolysaccharides (LPS) reduction after Sal treatment. *In vitro*, Sal showed PPARγ-dependent anti-inflammatory effect in LPS-stimulated RAW264.7 cells. In summary, our results demonstrated that Sal might be an effective factor for UC treatment and its pharmacological value deserved further development.

## Introduction

Inflammatory bowel diseases (IBD), known as a chronic, nonspecific, and relapsing inflammation of the gastrointestinal, is highly prevalent in developed countries (Zhang et al., 2017a). Ulcerative colitis (UC), a major subtype of IBD, is characterized by diarrhea, abdominal pain, weight loss, and rectal bleeding (Zhang et al., 2017b). Even now, its exact etiology and pathogenesis are still filmy. The susceptibility of host, environmental factors, imbalance of cytokines, and the intestinal microflora are thought to be related with UC (Ordas et al., 2012; Neurath, 2014; Ungaro et al., 2017).

It is reported that nuclear factor-kappa B (NF-κB) and mitogen-activated protein kinase (MAPK) play important roles in UC development (Gupta et al., 2015; Zhang et al., 2017a; Almeer et al., 2018). Once activated, a large number of transcription factors induced pro-inflammatory cytokines release, such as IL-1β, IL-6, and TNF-α (Yadav et al., 2003). Excessive inflammation can cause damage to the intestinal tract, and long-term exposure may lead to colon cancer (Pekow et al., 2017). Peroxisome proliferator-activated receptor (PPARγ), a transcriptional factor with anti-inflammatory function, is down-regulated during IBD with increased levels of inflammation (Rahimian et al., 2016). In addition, increasing evidence demonstrates that genetic modification is vital in the pathogenesis and developments of IBD (Zhang et al., 2014). For example, mice deficient for NOD-like receptor family pyrin domain containing 3 (NLRP3) were less impressible to acute colitis induced by dextran sodium sulfate (DSS) (Zhang et al., 2017b). However, *IL-10* *^-/-^* mice are susceptible to IBD and show hyperinflammation (Gurung et al., 2015). Autophagy as an important intracellular process is involved in many chronic inflammatory diseases. It is vital to maintain cell homeostasis and to respond to stimuli (nutrient deprivation, hypoxia, and oxidative stress) (Retnakumar and Muller, 2019). Since the ascertainment of the autophagy-related gene (ATG) 16L1 as a primary factor in IBD in 2006 (Hampe et al., 2007), the undiscovered mechanism between autophagy and IBD is becoming increasingly clear.

IBD patients show alterations in gut microbiota, such as growing numbers of pro-inflammatory and enteroadherent bacterial species, reduced diversity of microorganisms (Kelly and Ananthakrishnan, 2019), but whether these changes are the cause or the result of the disease remains unknown. Due to the destruction of intestinal mucosa, a large amount of LPS absorbed into the blood continuously stimulates the immune system of the body contributing to colitis (Chung et al., 2011; Kim et al., 2012). Colonic tight junction (TJ) proteins have been shown to adjust the LPS transfer from the intestinal tract into the blood (Park et al., 2010). Hence, maintaining the expression of TJ proteins can reduce LPS into the blood and alleviate inflammation (Trivedi and Jena, 2013).

Most therapeutic drugs can relieve clinical symptoms, however, prolonged treatment, side effects, and expensive cost are not the best choice for the most people. Salidroside (Sal), a major glycoside extracted from *Rhodiola rosea* L., has been proven to possess multiple pharmacological effects such as anti-aging, anti-oxidant, anti-cancer, anti-inflammation, and neuroprotective effects (Chen et al., 2009; Zhu et al., 2011; Gao et al., 2016). Yet, there is little information about the impact of Sal on UC. In this study, we explored the protective effects and illuminated the underlying mechanisms of Sal in the treatment of DSS-induced colitis.

## Materials and Methods

### Ethics Statement

All animal experiments were performed in strict accordance with regulations of the Administration of Affairs Concerning Experimental Animals in China. The protocol was approved by the Institutional Animal Care and Use Committee of Jilin University (20170318).

### Materials

Sal was obtained from TCI Chemical Industry Co., Ltd. (Shanghai, China). DSS (molecular weight of 36–50 kDa) was purchased from MP Biomedicals (Irvine, CA, USA). The primary antibodies p38, p-p38, p65, p-p65, and the secondary antibody horseradish peroxidase (HRP)-conjugated goat anti-rabbit antibody were purchased from Cell Signaling Technology, Inc. (Beverly, MA, USA). The secondary antibody HRP-conjugated goat anti-rabbit and goat anti-mouse antibody were obtained from Immunoway (Immunoway Technology, USA). The primary antibodies occludin and zonula occludens-1 (ZO-1) were purchased from Santa Cruz (Santa Cruz, CA, USA). β-Actin were purchased from Tianjin Sungene Biotech Co., Ltd. (Tianjin, China). All enzyme-linked immunosorbent assay (ELISA) kits were obtained from BioLegend (San Diego, CA, USA). Protein Extraction Kit was provided by Thermo Scientific Life Science Research (MA, USA). All other chemicals were of reagent grade.

### Animals

Male C57BL/6 mice (21-23 g) were provided from the Center of Experimental Animals of Jilin University, China. Before experiment, it takes 1 week for mice to adapt to new condition (24 ± 1°C).

### Dextran Sulfate Sodium-Induced Mice Colitis Model and Treatment

**Figure 1:**
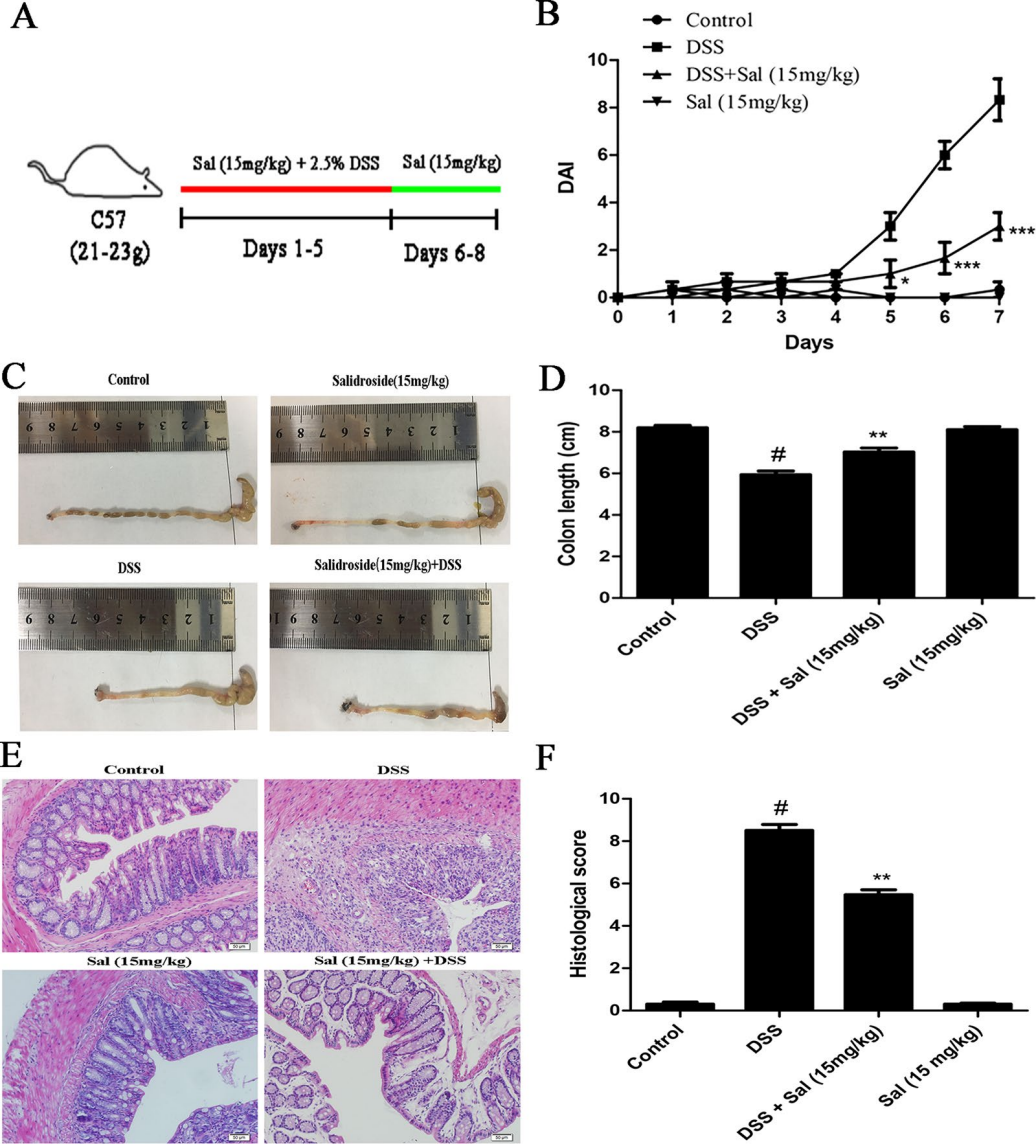
Salidroside alleviated dextran sulfate sodium (DSS)-induced colitis in mice. **(A)** The schematic diagram of DSS-induced colitis in mice treated with Sal (15 mg/kg). **(B)** Disease activity index (DAI) during the experimental process. **(C)** and **(D)**, the photos and statistical results of colon length, respectively. **(E)** Histopathological section of colon from each experimental group (200×; scale bar, 50 μm). **(F)** Histopathological scores of each group were calculated. Data were presented as the means ± SD. (*) p < 0.05, (**) p < 0.01, and (***) p < 0.001 *vs*. the DSS-treated group; (^#^) p < 0.05 *vs*. the control group.

Mice were randomly divided into four groups of six mice each. Acute colitis was induced by feeding mice with 2.5% (w/v) DSS, continuously for 5 days (Figure 1A). Mice in group I received water only. Mice in group II received 2.5% DSS in drinking water. Mice in groups III received Sal orally (15 mg/kg) for 7 days including 5 days DSS treatment once per day. Mice in group IV only received Sal (15 mg/kg). Body weights were measured once a day. The disease activity index (DAI) was assessed in line with established scoring system (Kihara et al., 2003). At the end of the experiment, mice were sacrificed, and the colon was excised from cecum to 1 cm above the anus. The colon specimens were fixed in 10% formalin for hematoxylin and eosin (H&E). Histological scoring was performed according to a method described previously and was determined by two independent, blinded investigators (Kihara et al., 2003; Hausmann et al., 2007). In brief, epithelium 0: normal morphology; 1: loss of goblet cells; 2: loss of goblet cells in large areas; 3: loss of crypts; 4: loss of crypts in large areas. Infiltration 0: no infiltrate; 1: infiltrate around crypt basis; 2: infiltrate reaching to *lamina muscularis mucosae*; 3: extensive infiltration reaching the *lamina muscularis mucosae* and thickening of the mucosa with abundant edema; 4: infiltration of the *lamina submucosa*. The final histological score is the sum of the epithelium and infiltration score.

### Cell Culture and Treatment

The RAW264.7 cells were cultured in RPMI-1640 medium supplemented with 10% fetal bovine serum, 100 U/ml penicillin, and 100 U/ml streptomycin at 37°C. And bone marrow derived macrophages (BMDMs) were isolated from mouse bone marrow and cultured in 10% macrophage colony-stimulating factor-containing RPMI 1640 medium supplemented with 10% fetal bovine serum for 7 days as previously described (Toma et al., 2011). Cells were pretreated in the presence or absence of Sal added 1 h prior to LPS (1 µg/ml). The supernatant and cell lysate were collected for cytokine detection and western blot, respectively. The PPARγ inhibitor GW9662 (10 µM) was added 30 min before Sal treatment.

### Enzyme-Linked Immunosorbent Assay

Homogenate of colon (100 mg) were prepared with phosphate-buffered saline (PBS) (w/v 1/9). The method of cytokine detection was performed with the instructions of the manufacturer (BioLegend, CA, USA).

### Western Blotting Analysis

Total proteins of cells and colon samples were extracted per the manufacturer’s protocol. And colon samples were selected randomly from three mice out of six. Quantified proteins were transferred onto polyvinylidene fluoride membranes. Membranes were blocked with 5% nonfat milk for 2 h, then they were incubated with the primary antibody overnight at 4°C. The second day, membranes were incubated with the secondary antibodies for 2 h at room temperature. The blots were examined with a western blotting detection program.

### Immunohistochemistry

Occludin and ZO-1 levels were detected according to the manufacture of immunohistochemistry (IHC) kit (Maixin, China). In brief, paraffin-embedded slides were deparaffinized, rehydrated, and washed with 1% PBS. Then, slides were hatched with 3% hydrogen peroxide and blocked with goat serum at 37°C for 1 h. After that, slides were incubated with primary antibodies (1:400) at 4°C. Slides were then transferred to PBS containing biotinylated secondary antibody for 1 h at room temperature. Streptavidin-HRP conjugates to the secondary antibody before treating with diaminobenzidine. Finally, slides were counter-stained with hematoxylin. Images were examined with a microscope (Olympus, Japan).

### Measurement of Lipopolysaccharides

The LPS of plasma was measure by limulus amebocyte lysate (LAL) assay kit (Lengton Bioscience Co., Ltd, Shanghai, China). Briefly, plasma was diluted with pyrogen-free water [1:9 (v/v)]. It was inactivated for 10 min at 70°C and incubated with LAL at 37°C for 30 min. Results were detected at 545 nm wavelength.

### Statistical Analysis

All data are shown as the mean ± standard deviation (SD). The differences of body weight and DAI were evaluated by the unpaired two tailed Student’s t-test. Data sets were measured by one-way ANOVA followed by Tukey’s multiple-comparison test. All experiments were repeated in triplicate. *P* < 0.05 was regarded statistically significant.

## Results

### Salidroside Alleviated Dextran Sulfate Sodium-Induced Colitis

DAI reflects the degree of injury of UC. As shown in Figure 1B, the DSS-treated mice exhibited a higher DAI scores than other groups, yet, Sal treatment significantly lowered the scores. The shortened colon is an indirect reflection of the pathological course of colitis. Sal administration remitted DSS-induced colonic shortening (Figures 1C, D). Meanwhile, histopathologic images demonstrated that mice in DSS group presented prominent acute inflammatory reaction accompanying by loss of goblet cells, hyperemia, and mucosal injury. These symptoms were relieved in DSS-treated mice after Sal administration (Figures 1E, F).

### Salidroside Ameliorated Dextran Sulfate Sodium-Induced Inflammation in Colonic Tissues

**Figure 2:**
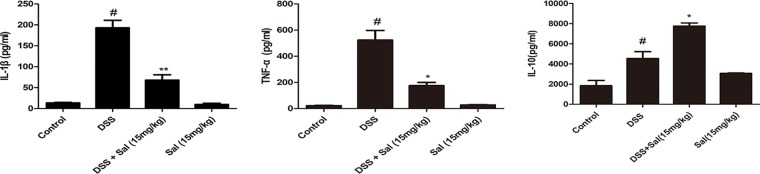
Salidroside adjusted the inflammatory conditions in dextran sulfate sodium (DSS)-treated mice. IL-1β, TNF-α, and IL-10 protein level in the colonic tissue were detected by enzyme-linked immunosorbent assay. Data were presented as the means ± SD. (*) p < 0.05 and (**) p < 0.01 *vs*. the DSS-treated group; (^#^) p < 0.05 *vs*. the control group.

The protein level of IL-1β, TNF-α, and IL-10 were markedly up-regulated after DSS treatment in mice, apparently, Sal suppressed IL-1β and TNF-α production in contrast to IL-10 in colonic tissues (Figure 2).

### Salidroside Inhibited p38 and p65 Activations and Promoted Peroxisome Proliferator-Activated Receptor Expression in Colonic Tissues

**Figure 3:**
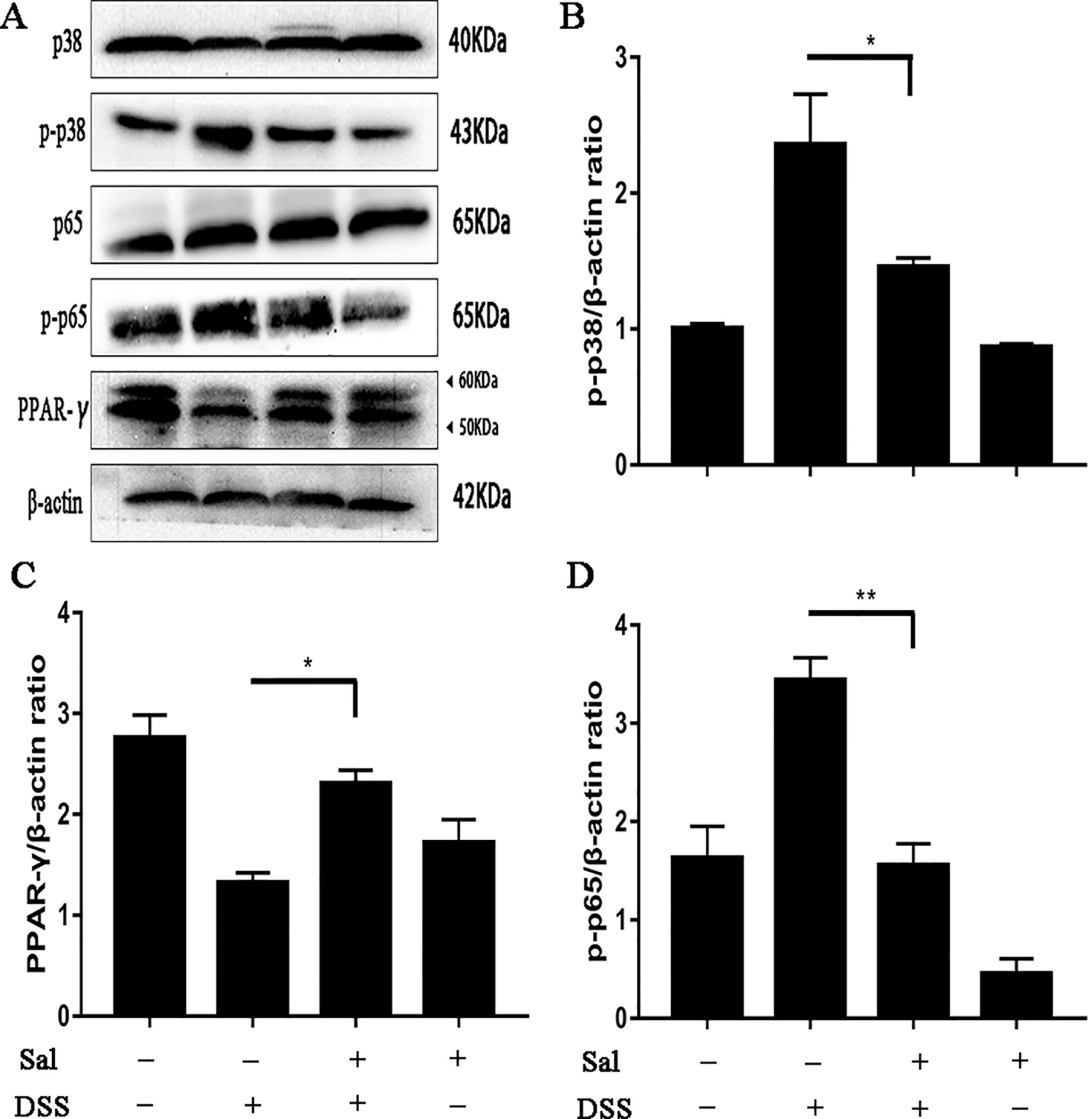
Salidroside restrained the phosphorylation of p65 and p38 accompanied with recovering peroxisome proliferator-activated receptor (PPARγ) protein level in dextran sulfate sodium-treated mice. **(A)** P65, p38, and PPARγ protein levels in the colonic tissue were analyzed by western blot. **(B, C, D)** The relative protein expression of p65, p38, and PPARγ were normalized to β-actin. Data were repeated in three independent experiments. (*) p < 0.05, (**) p < 0.01.

To explore the mechanism of inflammatory level alteration, we detected the expression of MAPK p38 and NF-κB p65 by western blot. The results showed that DSS treatment significantly up-regulated the phosphorylation levels of p38 and p65 in colonic tissues, however, this situation was inhibited in Sal-treated group. It was reported that PPARγ played a satisfactory anti-inflammatory role in experimental colitis models (Cao et al., 2018). Comparing with the control group, PPARγ expression was remarkable reduced in the DSS group. But Sal treatment increased PPARγ level compared with the DSS group (Figure 3).

### Salidroside Suppressed Nucleotide Oligomerization Domain-Like Receptor Family Pyrin Domain Containing 3 Inflammasomes in Colonic Tissues

**Figure 4:**
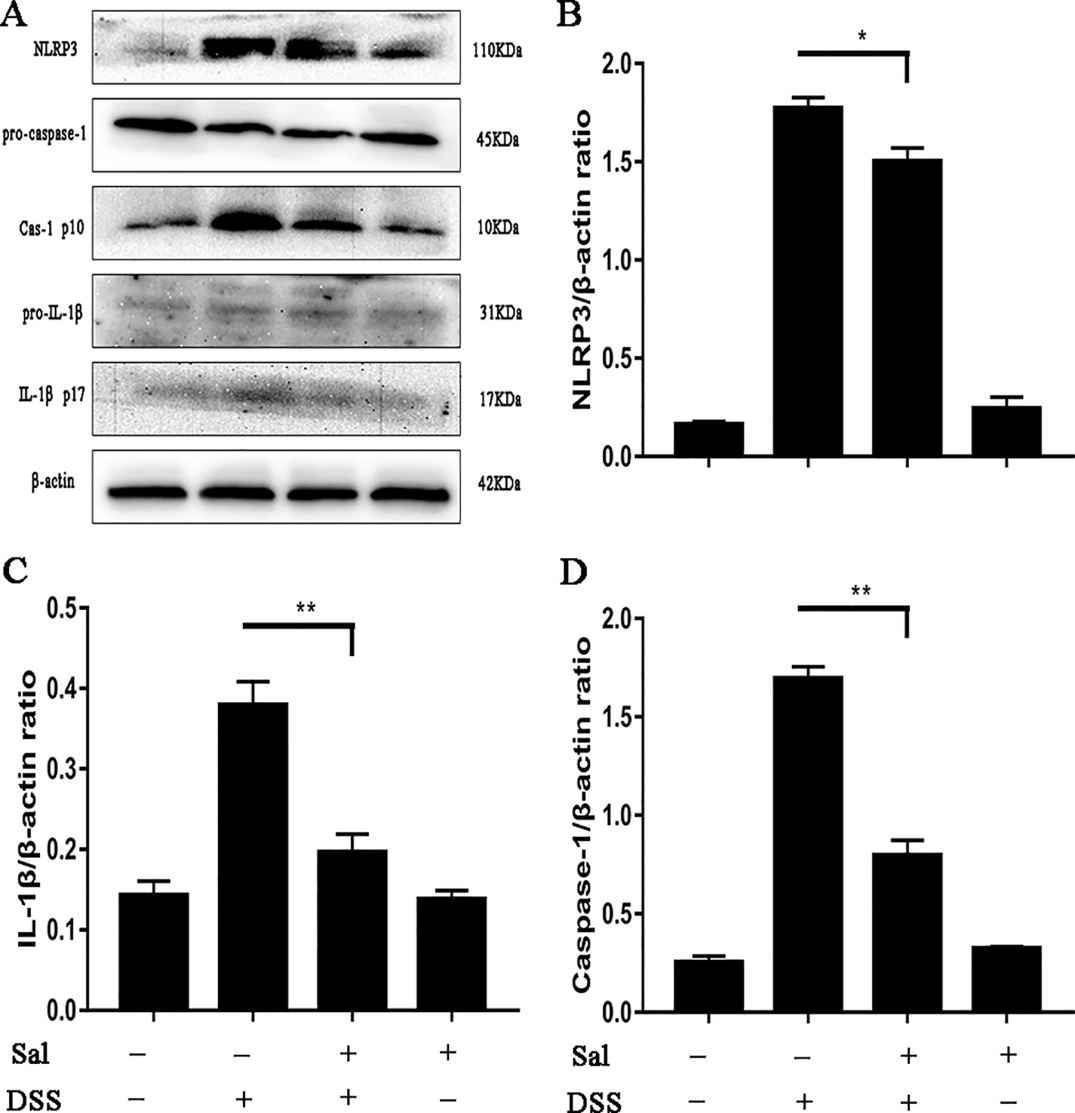
Salidroside suppressed nucleotide oligomerization domain-like receptor family pyrin domain containing 3 (NLRP3) inflammasome activation in dextran sulfate sodium-treated mice. **(A)** Protein levels of NLRP3, caspase-1, and IL-1β in the colonic tissue were analyzed by western blot. **(B, C, D)** The relative protein expression of NLRP3, caspase-1, and IL-1β were normalized to β-actin. Data were repeated in three independent experiments. (*) p < 0.05, (**) p < 0.01.

The release of IL-1β is controlled by NLRP3 inflammasome, but NLRP3 inflammasomes activation is regulated by two phases, including priming phase and activation phase (Di et al., 2018). The results showed Sal administration down-regulated the protein level of IL-1β in colonic tissues (Figure 2). So, we wondered to know which pathway did Sal affect, or both of two. The results showed that Sal treatment inhibited the priming and activation of NLRP3 inflammasome compared with the DSS group (Figure 4).

### Salidroside Altered the Level of Autophagy in Colonic Tissues

**Figure 5:**
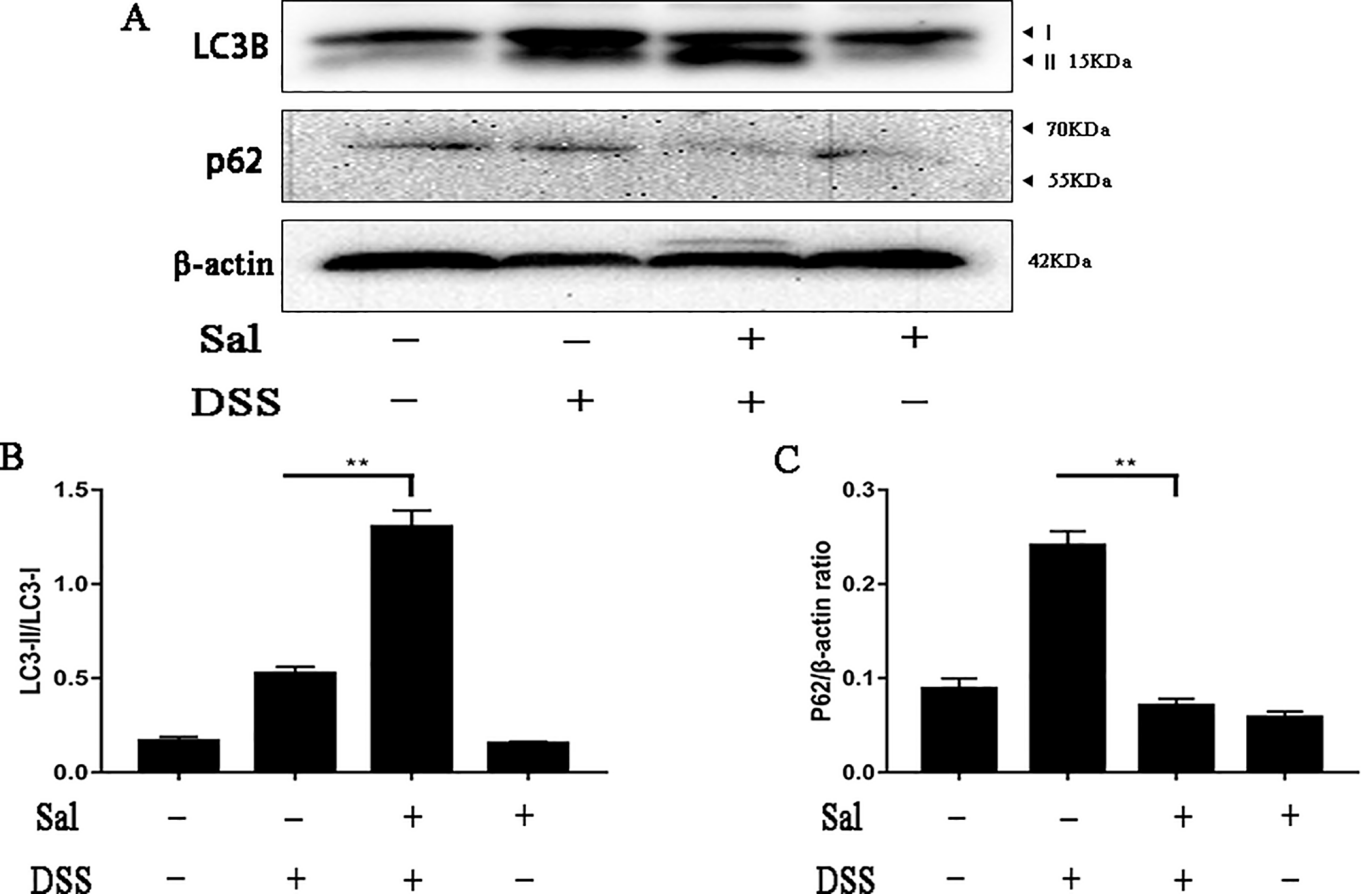
Salidroside regulated the disordered autophagy situation in dextran sulfate sodium-treated mice. **(A)** Protein levels of LC3 and p62 in the colonic tissue were analyzed by western blot. **(B, C)** The relative protein expression of LC3 and p62 were normalized to β-actin. Data were repeated in three independent experiments. (**) p < 0.01.

Autophagy has been reported to be crucial in IBD process (Retnakumar and Muller, 2019), we aimed to explore the effect of Sal on autophagy during DSS-induced colitis. Sal promoted significantly the conversion of LC3-I to LC3-II and reduced p62 expression detecting by WB (Figure 5).

### Salidroside Maintained the Intestinal Barrier in Dextran Sodium Sulfate-Induced Colitis

**Figure 6:**
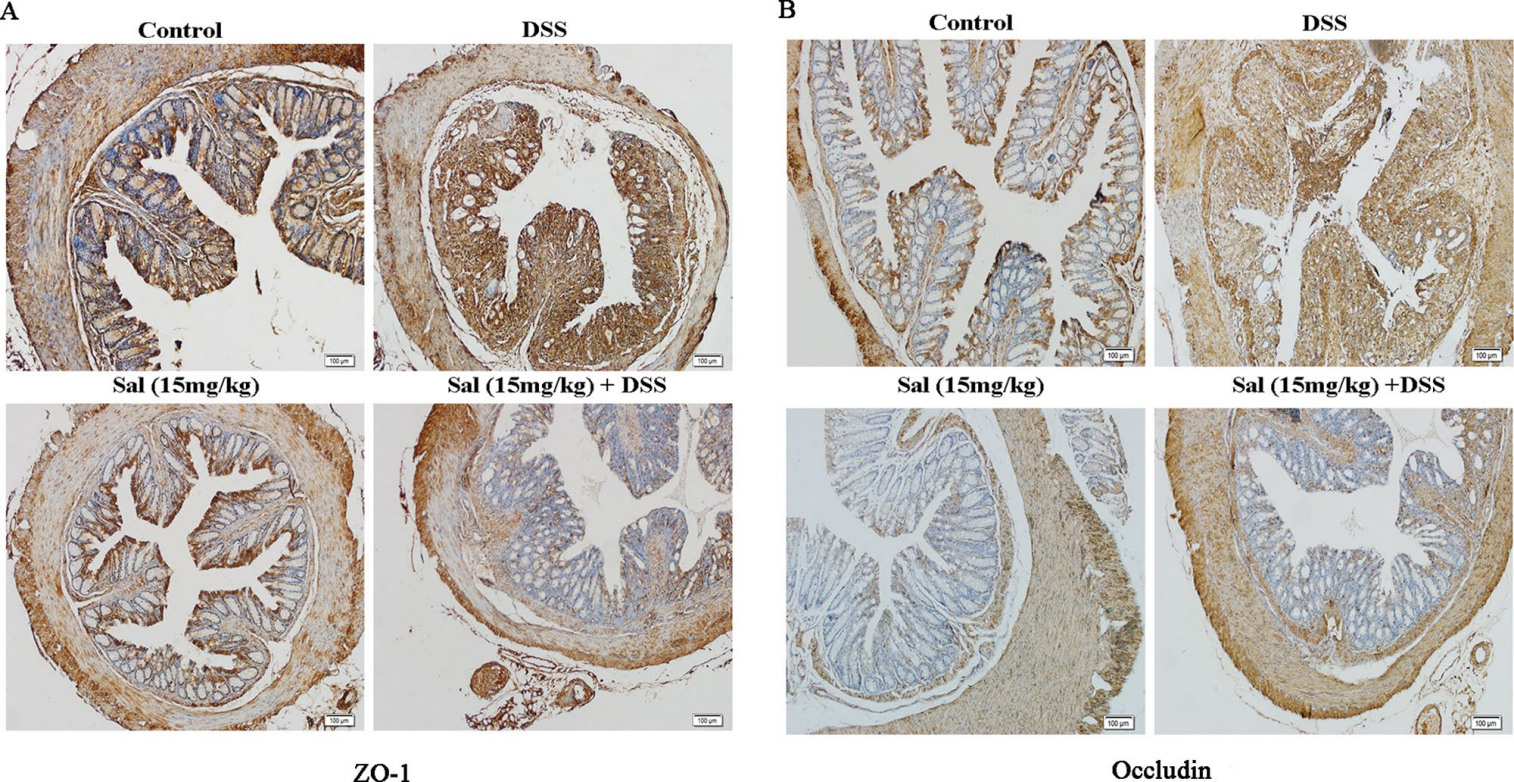
Salidroside contributed to the maintenance of tight junction architecture in dextran sulfate sodium-treated mice. **(A)** and **(B)** Immunohistochemistry for zonula occludens-1 and occluding in the colonic tissue, respectively. (400×; scale bar, 100 μm).

Abnormalities of the mucus system have been introduced in active UC, and the changes in intestinal permeability are an important factor. In our study, DSS-treated mice exhibited insufficient expression of ZO-1 and occludin in colonic tissues analyzed IHC (Figure 6), while Sal treatment ameliorated these situations (Figure 6).

### Salidroside Reduced Plasmatic Lipopolysaccharides in Dextran Sulfate Sodium-Induced Mice

**Figure 7:**
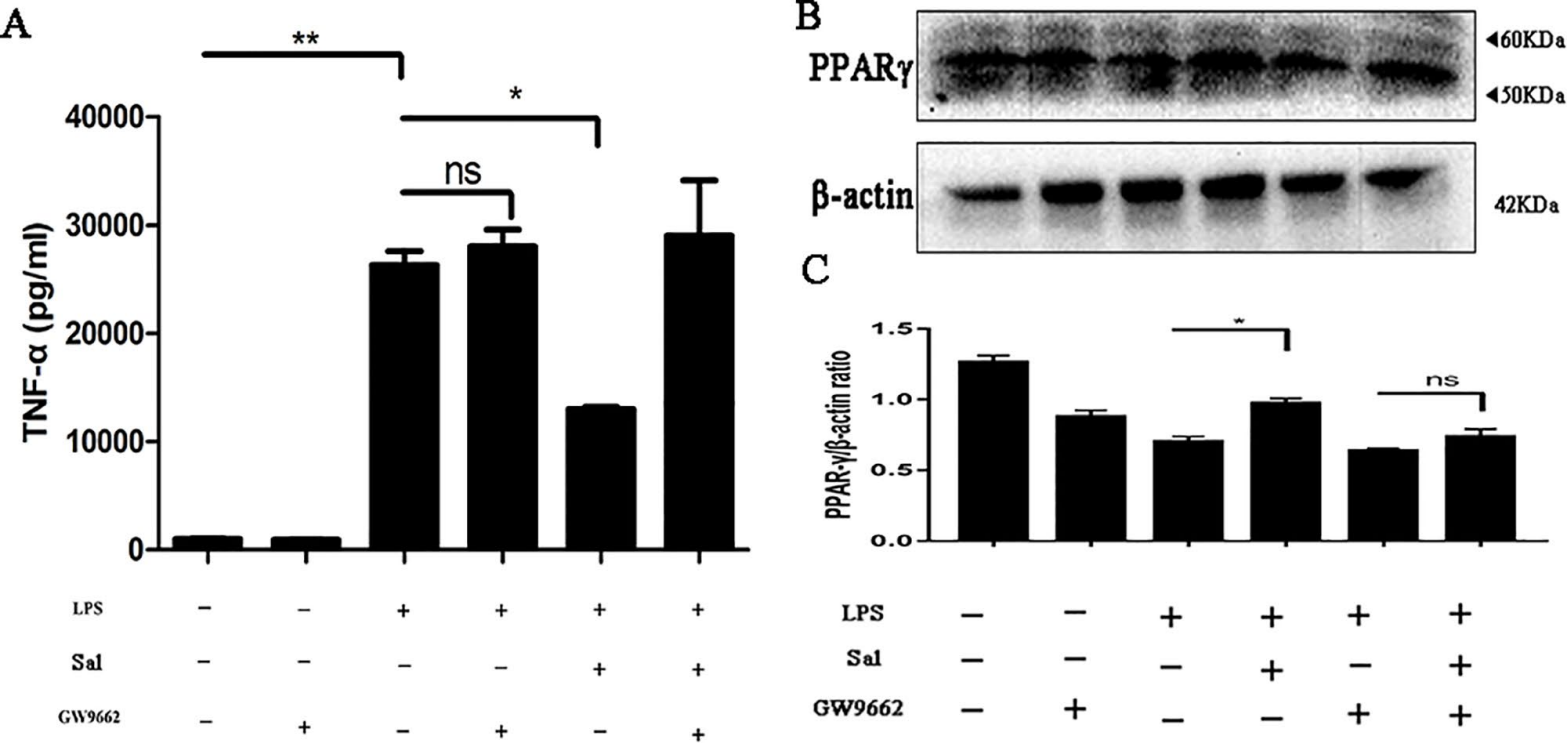
Salidroside exhibited anti-inflammatory activity through regulating peroxisome proliferator-activated receptor (PPARγ) expression. **(A)** The protein level of TNF-α was measured by enzyme-linked immunosorbent assay. **(B)** The expression of PPARγ was analyzed by western blot. **(C)** The relative protein expression of PPARγ was normalized to β-actin. Data were repeated in 3 independent experiments. (*) p < 0.05, (**) p < 0.01, ns, means no significant.

LPS absorbed into the blood continually stimulates the immune system to promote inflammation. To further study the effect of Sal on DSS-induced colitis, we detected plasmatic LPS concentration in mice. As shown in Figure 7, LPS concentration was evidently higher in DSS group than that in control group. However, Sal prominently lowered LPS concentration compared with DSS group.

### Salidroside Showed Peroxisome Proliferator-Activated Receptor-Dependent Anti-Inflammatory Effect on Macrophages

Macrophages are a major source of inflammation in colon, and excessive inflammation can lead to tissue damage. Hence, we explored the anti-inflammatory effect of Sal on LPS-treated RAW 264.7 cells. To further confirm the effect of Sal on macrophages *in vitro*, we did the same procedure on BMDM. The results demonstrated that Sal was able to reduce TNF-α protein level (Figure 7A and **Supplementary Figure 1**) and it worked through PPARγ (**Figure 7B**).

## DISCUSSION

UC, a kind of chronic and recurrent disease of gastrointestinal tract, is considered to be related to environment, genetics, host susceptibility, immune level, and intestinal microflora (Retnakumar and Muller, 2019; Shen et al., 2019). It has a negative impact on millions of people worldwide, with the highest incidence in Europe and North America. Moreover, it also has a rising prevalence in newly industrialized countries, probably due to diet, lifestyle, and sanitation (Kaplan, 2015; Retnakumar and Muller, 2019). The current treatments are mainly used with anti-inflammatory or immunosuppressive drug, however, these agents are expensive and take longer to treat than most people can afford (Zhang et al., 2017a). At present, the priority is to find a cheap and side-effect-free drug to treat UC. Sal, as a major glycoside extracted from *R. rosea* L., showed its anti-oxidant, anti-cancer, anti-inflammation, and neuroprotective effects in many diseases (Chen et al., 2009; Zhu et al., 2011; Gao et al., 2016). In this study, we exploited its new features against UC and investigated the underlying mechanisms.

DAI and length of the colon are the primary parameters used for assessing the severity of UC (Zhang et al., 2017b). Our results demonstrated that Sal could significantly decrease DAI scores and remit colon shortening in DSS-treated mice (**Figures 1B–D**). In addition, Sal treatment also relieved DSS-induced goblet cell loss and twisted crypts analyzed by histopathological examination (**Figures 1E, F**). It was reported that goblet cell consuming was related with an infernal circle of increased epithelial exposure to bacteria, worsening inflammation, and mucus barrier breakdown (van der Post et al., 2019). Hence, taking all of these parameters together, Sal can be considered as a potent candidate for treatment of UC.

The development of UC is often accompanied by increased levels of inflammation. Once the mucosal barrier is impaired, intestinal epithelial cells release cytokines that recruit large numbers of immune cells and cause excessive inflammation (Zhang et al., 2017a; van der Post et al., 2019). Obviously, Sal down-regulated the secretion of IL-1β and TNF-α, but promoted the level of IL-10 (**Figure 2**). As is known to all, IL-1β and TNF-α are representative cytokines that aggravates colitis (Nakanishi et al., 2015), the down-regulation of these cytokines caused by Sal treatment in DSS-induced colitis mice might help ease disease. In addition, IL-10 is a pleiotropic anti-inflammatory cytokine produced by most hematopoietic cells. Genome-wide association studies and experimental animal models showed an essential role of the IL-10 in inflammatory bowel disease (Zigmond et al., 2014). The up-regulation of IL-10 could reduce pro-inflammatory cytokines which mediated its potential in medical therapy for colitis (Kiernan et al., 2019; Jang et al., 2019). NF-κB and MAPK are two vital regulators participating in regulating inflammation (Kim et al., 2010; Cao et al., 2018). Our result showed that DSS-induced activation of p65 and p38 were inhibited by Sal (**Figure 3**). Looking further up for targets of Sal, it was reported that PPARγ was an essential anti-inflammatory factor through inhibiting the activation of NF-κB signal in the process of colonic inflammation (Zhang et al., 2017a). It could interact with p65 and promote p65 out of nuclear leading to the inactivation of NF-κB-driven transcription (Cao et al., 2018). The protein level of PPARγ in DSS-treated group was elevated after Sal administration (**Figure 3**). Consistent with the result obtained *in vitro* that Sal revealed its anti-inflammatory trait in LPS-treated macrophage in a PPARγ-dependent way (**Figure 7** and **Supplementary Figure 1**). Hence, we inferred that Sal might regulate colonic inflammation in an axis-dependent manner by PPARγ-NF-κB and MAPK.

As is known to all, NLRP3 inflammasome was strongly associated with IBD. *NLRP3* *^-/-^* mice showed less sensitive to colitis (Zhang et al., 2017b; Shen et al., 2019). Not only that, it also tightly controlled the production of IL-1β, which was considered as one of the important indicators of colon inflammation (Zhang et al., 2017b). Of course, the very mention of inflammasomes brings to mind autophagy (Cosin-Roger et al., 2017). There were few studies on Sal and inflammasome, Wang et al. reported that Sal could alleviate ventilation induced lung injury by suppressing NLRP3 inflammasome (Wang et al., 2017). This suggested that Sal might have the same function in UC. Simultaneously, previous study demonstrated that Sal was potent in inducing apoptosis and autophagy in human colorectal cancer cells, which was a further result of colitis (Fan et al., 2016; Zhang et al., 2017a). In this study, we investigated the potential pharmacological targets of Sal and relationship of NLRP3 inflammasome and autophagy in UC. Our results showed that Sal inhibited NLRP3 inflammasome activation by down-regulating the levels of NLRP3, apoptosis-associated speck-like protein (ASC), and caspase-1 in DSS-treated group (**Figure 4**). So, it was not hard to understand why IL-1β secretion was lower in DSS-treated mice after Sal treatment (**Figure 2**). P65/RelA could bind to the NLRP3 promoter (Cosin-Roger et al., 2017), hence, this is consistent with our result above that the phosphorylation of p65 was restrained in Sal-treated DSS group (**Figure 3**). In contrast with the status of NLRP3 inflammasome in DSS-treated mice after Sal administration, autophagy might be activated with LC3 lipidation (LC3-II) and p62 degradation (**Figure 5**). Certainly, the exact autophagy flux should be further determined by autophagy inhibitor (chloroquine or bafilomycin A) or the formation of LC-3 puncta. Autophagy insufficient was related with NF-κB phosphorylation and inflammatory gene expression in IBD (Kaser and Blumberg, 2011). Autophagy activation contributed to removing endogenous inflammasome activators, inflammasomes, and downstream cytokines directly (Sun et al., 2017). Therefore, Sal may inhibit excessive inflammation caused by inflammasome activation through autophagy excitation. However, the exact target of Sal on autophagy and inflammasome is worth further investigation.

**Figure 8:**
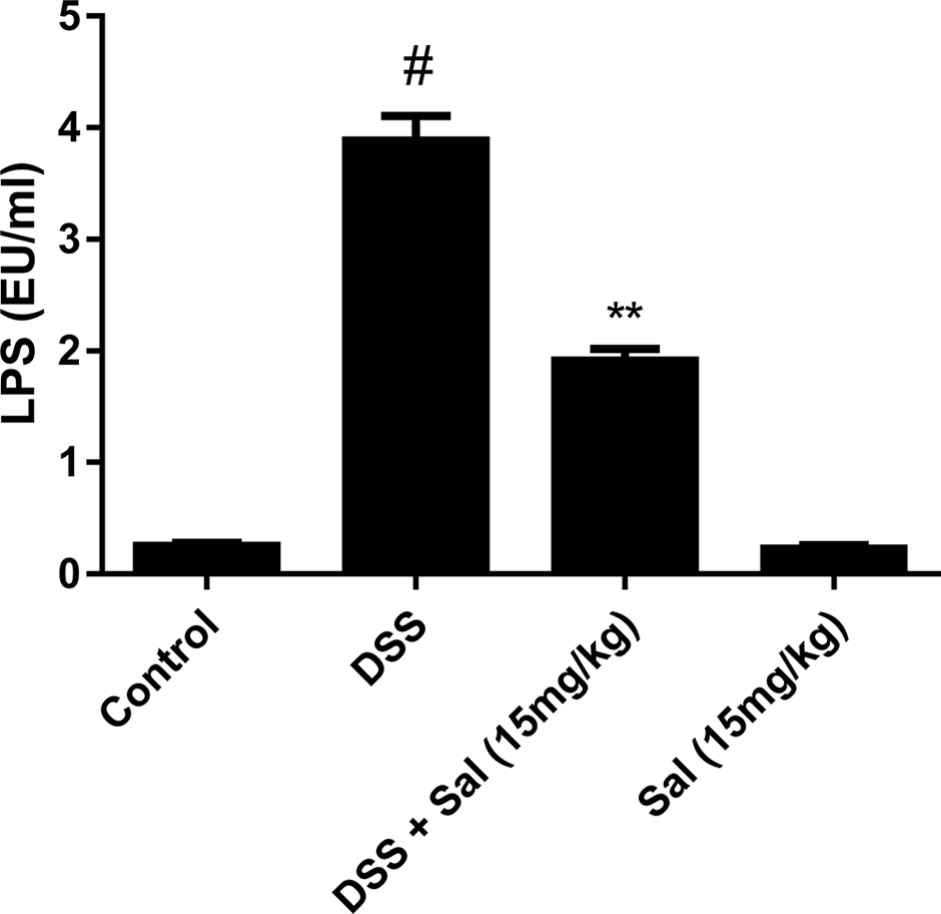
Salidroside reduced plasmatic lipopolysaccharides (LPS) concentration in dextran sulfate sodium (DSS)-treated mice. Limulus amebocyte lysate assay was used to assess the plasmatic LPS concentration. Data are presented as means ± SD. (*) p < 0.05 *vs*. the DSS-treated group; (^#^) p < 0.05 *vs*. the control group.

The development of IBD is facilitated by the destruction of the intestinal environment and improper activation of the immune system by commensal bacteria, which is the basis of the disease (van der Post et al., 2019). Increased epithelial exposure to bacteria may cause severe colitis to progress to colon cancer. TJ proteins as an essential component of the intestinal barrier, are able to reduce intestinal permeability and inhibit the intestinal mucosa of foreign substances, such as LPS (Zhang et al., 2017b). Our results reveled that Sal ameliorated DSS-induced suppression of TJ proteins, ZO-1 and occludin (**Figure 6**) and plasma LPS (**Figure 8**). It indicated that Sal might play an important role in maintaining intestinal barrier and reducing external stimuli. Given the structural weakening of the colonic mucus barrier is an early time for UC (van der Post et al., 2019), the detailed protective mechanism of Sal on colon is worth further exploration.

In summary, our study demonstrated that Sal could be a competent candidate for UC treatment. It not only exhibited its powerful abilities to skew the unbalanced relation of inflammasome/autophagy and maintain intestinal barrier but also further uncovered the underlying mechanism of UC pathogenesis. We need to do more work to discover the properties of salidroside in the future.

## Data Availability Statement

All datasets generated for this study are included in the article/**Supplementary Material**.

## Ethics Statement

The animal study was reviewed and approved by the Institutional Animal Care and Use Committee of Jilin University (20170318).

## Author Contributions

JL analyzed the data and wrote the article. JC collected the samples and operated the experiment. PF operated the experiment and processed the data. NZ revised the article. YC designed the experiment and supervised the experiment,

## Funding

This work was supported by the Key Project of Chinese National Programs for Research and Development (no. 2016YFD0501009) and National Natural Science Foundation of China (nos. 31572582 and 31472248).

## Conflict of Interest

The authors declare that the research was conducted in the absence of any commercial or financial relationships that could be construed as a potential conflict of interest.

## Supplementary Material

Supplementary Figure 1 Sal could reduce TNF-α level in LPS-stimulated BMDM. Data were repeated in 3 independent experiments. (*) p < 0.05, (**) p < 0.01.
