# Supplementary figures and images for: The Abilities of Salidroside on Ameliorating Inflammation, Skewing the Imbalanced Nucleotide Oligomerization Domain-Like Receptor Family Pyrin Domain Containing 3/Autophagy, and Maintaining Intestinal Barrier Are Profitable in Colitis
Source: Front Pharmacol. 2019 Dec 2;10:1385. doi: 10.3389/fphar.2019.01385 (PMC6901016; doi:10.3389/fphar.2019.01385)

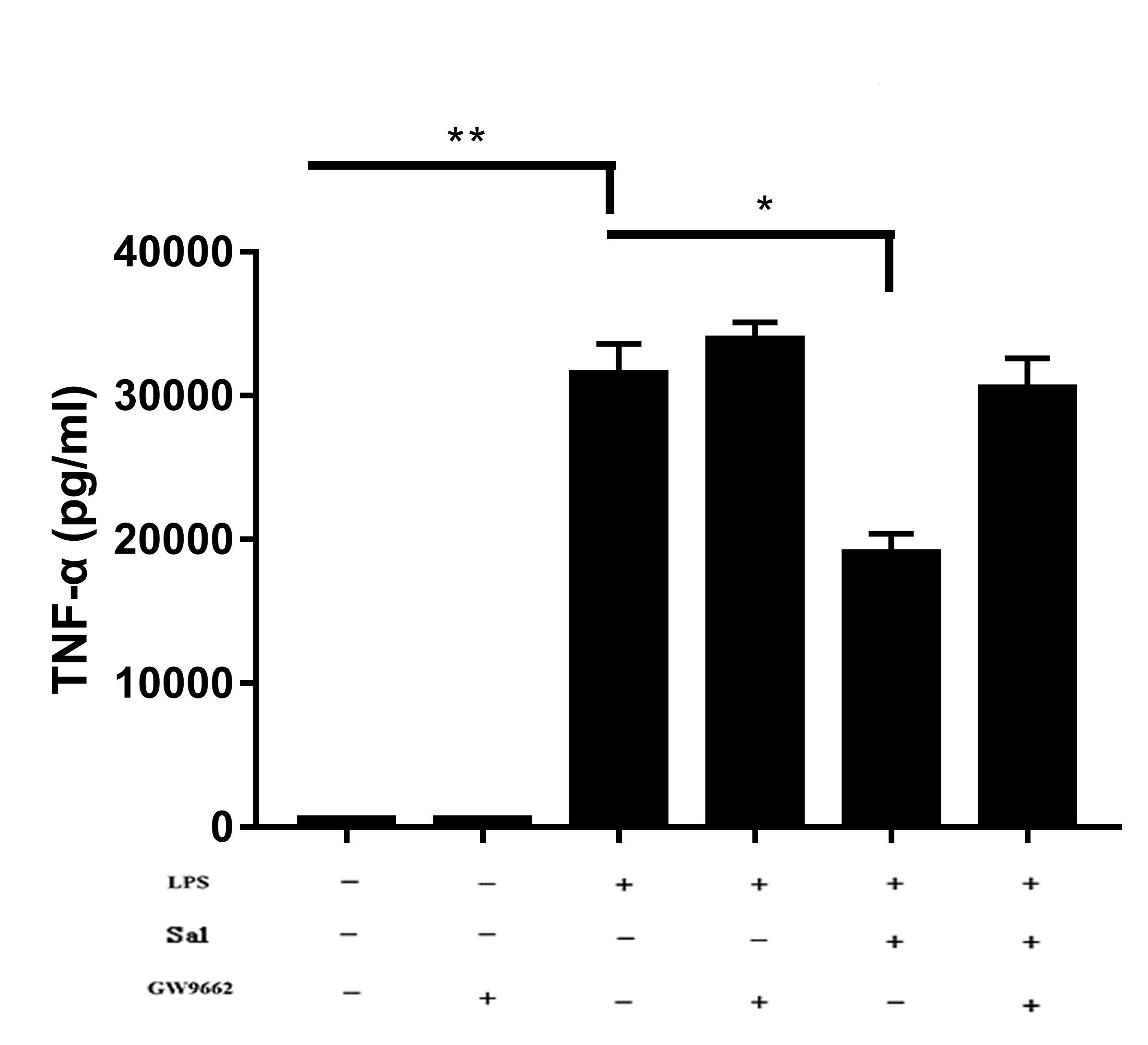

Supplement: Supplementary Figure 1 — Sal could reduce TNF-α level in LPS-stimulated BMDM. Data were repeated in 3 independent experiments. (*) p < 0.05, (**) p < 0.01. [file Image_1.tif]
